# Pembrolizumab-Induced Rupioid Psoriasis Treated by Apremilast Without Interrupting the Ongoing Antineoplastic Treatment

**DOI:** 10.7759/cureus.55249

**Published:** 2024-02-29

**Authors:** Francesca Ambrogio, Luca Rubino, Carmelo Laface, Gerardo Cazzato, Caterina Foti

**Affiliations:** 1 Department of Precision and Regenerative Medicine and Ionian Area, University of Bari Aldo Moro, Bari, ITA; 2 Medical Oncology, Dario Camberlingo Hospital, Francavilla Fontana, ITA

**Keywords:** dermatopathology, pembrolizumab, icis, rupioid, psoriasis

## Abstract

We report the case of a 77-year-old man affected by a poorly differentiated metastatic pulmonary adenocarcinoma who, after the first course of therapy with cisplatin-pemetrexed-pembrolizumab treatment, developed rupioid psoriasis. We decided to discontinue pembrolizumab for four weeks until lesions improved and to start therapy with apremilast (an oral small molecule phosphodiesterase (PDE)4 inhibitor) in combination with systemic methylprednisolone 16 mg/day with consequent tapering until discontinuation in a few weeks.

After accomplishing three months of treatment with apremilast, the patient gained complete remission of the rupioid lesions. Pembrolizumab therapy was reintroduced, and cycles were carried out without exacerbating the clinical picture. During the fourth month of therapy with apremilast, it was attempted to stop the treatment despite continuing the therapy with pembrolizumab. As a result, there was a relapse of the erythematous scaling plaques. After the subsequent reintroduction of apremilast, a new remission of the clinical picture occurred despite the absence of interruption of pembrolizumab. As far as we know, this is the second case of rupioid psoriasis induced by immunotherapy with pembrolizumab. Still, while the previous case was undergoing therapy with acitretin and methylprednisone, our patient is the first case treated with apremilast with excellent and rapid remission even after discontinuation and re-administration of pembrolizumab without exacerbation of dermatitis. In addition, the appearance of psoriasis during immunotherapy can be properly treated, which does not contraindicate the continuation of the antineoplastic treatment.

## Introduction

Pembrolizumab, an immune checkpoint inhibitor (ICI) targeting programmed death receptor-1 (PD-1), has increased the survival rate of patients affected by different types of cancers. It was approved by the United States Food and Drug Administration (US FDA) in 2015 and, over the years, has gained progressively more relevance in the field of immunotherapy for melanoma, non-small cell lung cancer, non-Hodgkin lymphoma, and so on. It is a humanized monoclonal antibody that binds the PD1 receptor expressed on T cells and blocks its interaction with programmed cell death ligand (PDL)1 and PDL2 ligands [1]. The PD1 receptor is a negative regulator of T cell activity, so blocking this interaction enhances the response of T cells to cancer cells. Because of their mechanism of action, immune checkpoint inhibitors like pembrolizumab relate to a unique toxicity profile that entails a broad clinical spectrum of immuno-related adverse events (irAEs). In general, skin toxicity is among the most common irAE [2]. This article was previously presented as an e-Poster at the European Academy of Dermatology and Venereology (EADV) Congress on 11-14 October 2023.

## Case presentation

**Figure 1:**
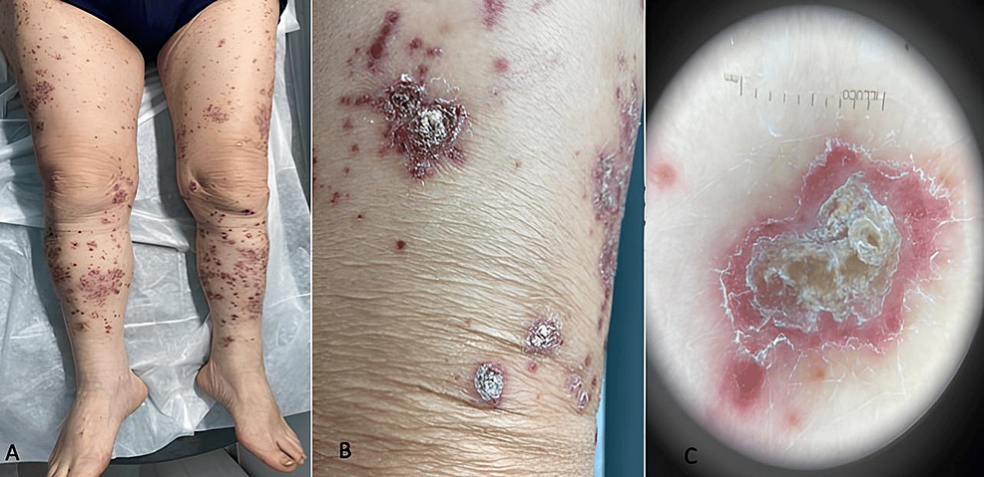
Patient images after the first course of treatment (A) and (B) Purpuric macules surmounted by cone-shaped hyperkeratotic plaques; (C) At the dermoscopy: the thick scales obscure the projection of the vascular pattern of psoriasis.

We report the case of a 77-year-old man affected by a poorly differentiated metastatic pulmonary adenocarcinoma undergoing therapy with cisplatin 75 mg/m^2^-pemetrexed 500 mg/m^2^-pembrolizumab 200 mg q21 days. After the first course of treatment (21 days), the patient developed erythematous and purpuric macules surmounted by scales giving plaques with rupioid appearance (Figures 1A-1B) with typical dermoscopy (Figure 1C) affecting his trunk, scalp, upper limbs, and lower limbs. He showed adherent keratotic lesions with reddish, sharply demarcated borders and oyster or limpet shell-shaped (Figures 1A-1C). In contrast to the rupioid form, regular plaque-type psoriasis has a white, nonadherent, and thin, scaly surface. He had a positive family history of psoriasis from his mother.

**Figure 2:**
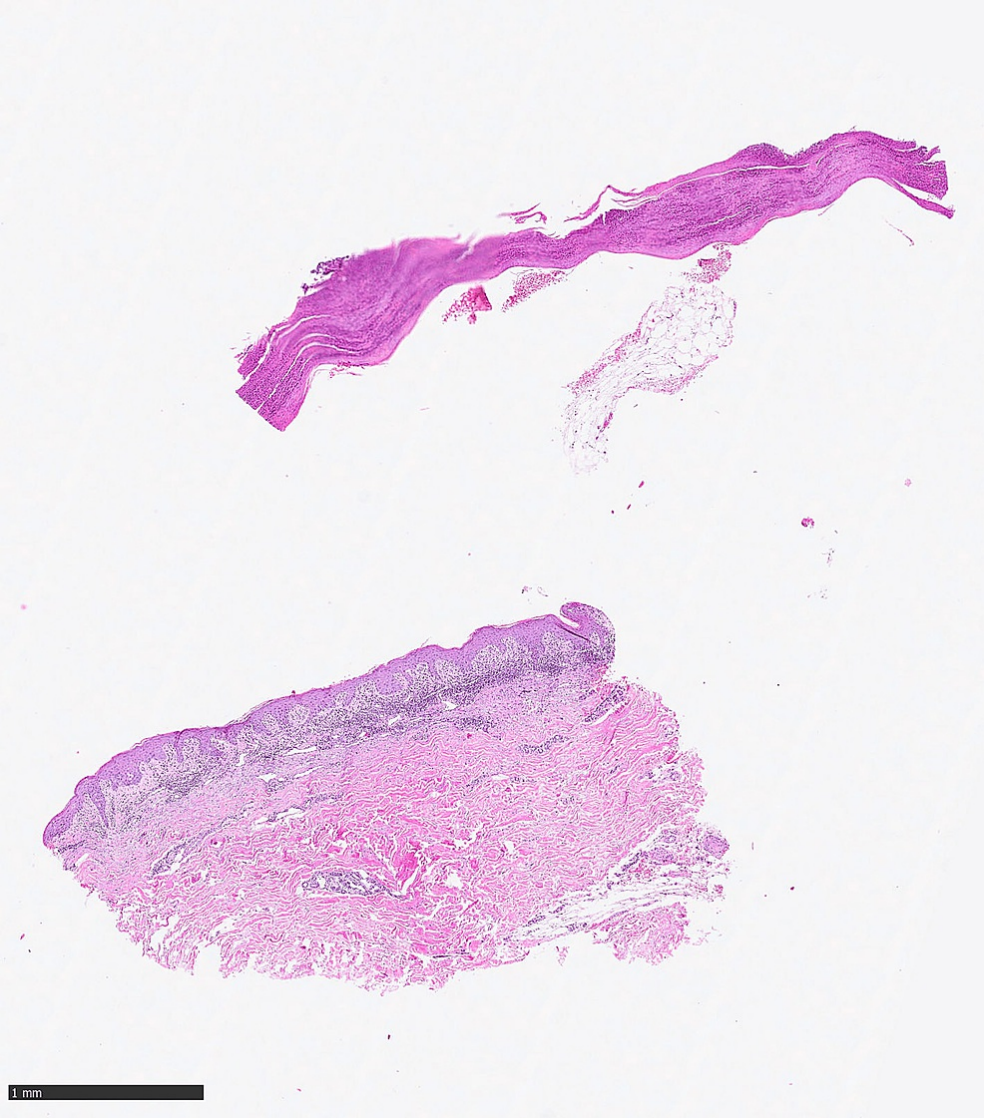
Histopathological features Skin biopsy showing compact parakeratosis with Munro microabscess, psoriasiform epidermal hyperplasia with fusion of contigue epidermal ridges and ascent of dermal microvessels.

In the suspicion of rupioid psoriasis, a skin biopsy was performed (Figure 2), which highlighted the histopathological features of a multi-layered parakeratosis, psoriasiform epidermal hyperplasia with a fusion of the contiguous epidermal ridges and curvy vessels, in accordance with our diagnostic hypothesis. Nails, scalp, face, palms, and mucosae were uninvolved. Laboratory testing was normal, including non-reactive syphilis and HIV serologies.

According to the literature, only one case of pembrolizumab-induced rupioid psoriasis has been described. After oncological consultation, we decided to discontinue pembrolizumab for four weeks until lesions improved and to start therapy with apremilast (an oral small molecule phosphodiesterase (PDE)4 inhibitor) in combination with systemic methylprednisolone 16 mg/day with consequent tapering until discontinuation in a few weeks.

**Figure 3:**
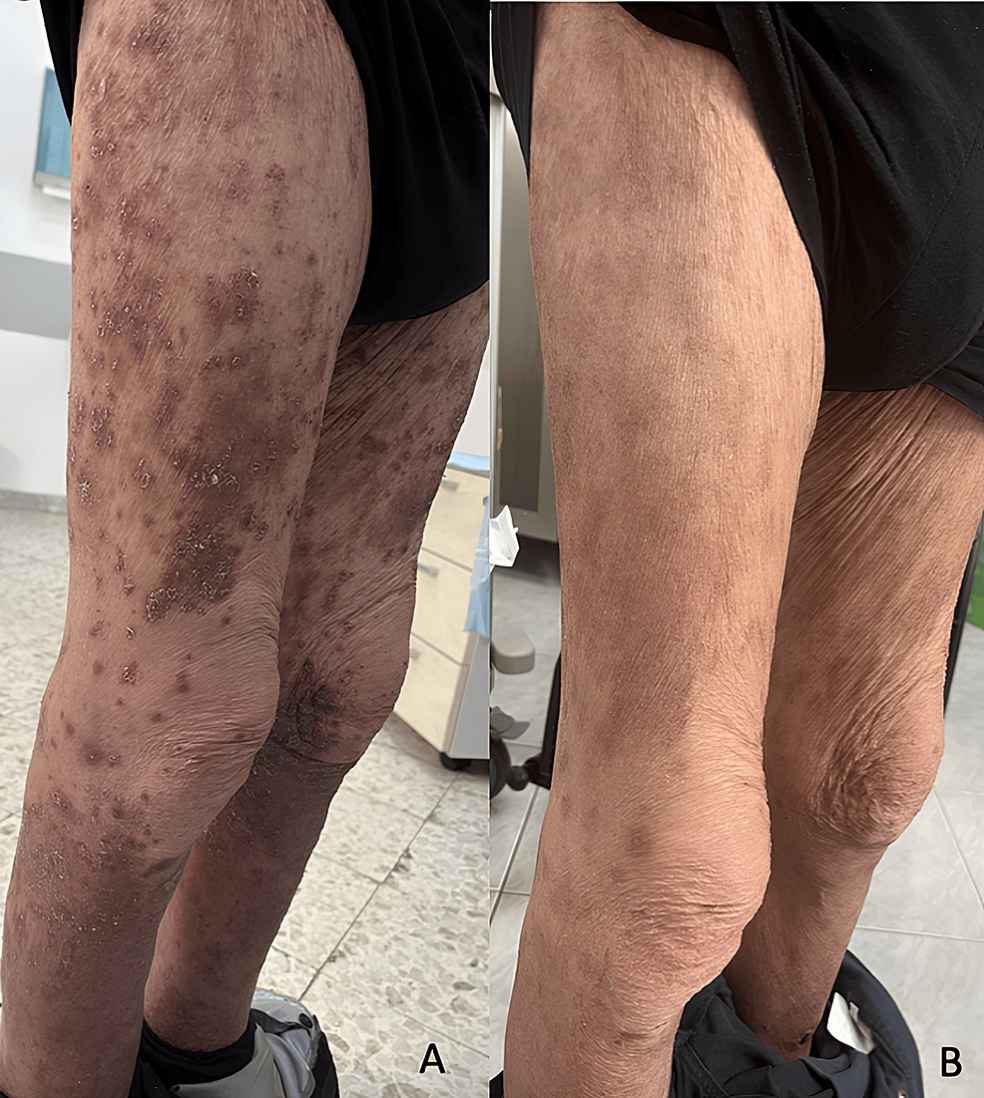
Patient images during the course of treatment (A) The relapse of erythematous scaling plaques; (B) Remission of the clinical picture with apremilast despite the absence of interruption of pembrolizumab.

After accomplishing three months of treatment with apremilast, the patient gained complete remission of the rupioid lesions. Pembrolizumab therapy was reintroduced, and cycles were carried out without exacerbating the clinical picture. During the fourth month of therapy with apremilast, it was attempted to stop the treatment despite continuing the therapy with pembrolizumab. As a result, there was a relapse of the erythematous scaling plaques (Figure 3A). After the subsequent reintroduction of apremilast, a new remission of the clinical picture occurred despite the absence of interruption of pembrolizumab (Figure 3B).

## Discussion

The term 'rupioid' is used to describe a well-defined, grossly cone-shaped, circular, thick keratotic lesion resembling the limpet shell or oyster shell [3-6]. This rupioid clinical aspect of the lesions can be observed in psoriasis, secondary syphilis, Norwegian scabies, HIV, sarcoidosis, reactive arthritis, disseminated histoplasmosis, or acrokeratosis paraneoplastica [4-6]. It's important to exclude acrokeratosis paraneoplastic condition, also known as Bazex syndrome, especially when erythematous rashes and psoriasiform scales are localized mostly in acral sites because it can be associated with squamous cell carcinoma of the aerodigestive tracts. Drug-provoked rupioid psoriasis (either drug-aggravated or drug-induced) is similarly infrequent. Lithium, beta-blockers, and antimalarials can be the most common causative drugs of psoriasis and also of rupioid type [6,7]. Various cases of treatment-induced psoriasis with ICIs (pembrolizumab, nivolumab) of different clinical types have been described in the literature, such as vulgaris [8], pustulate [9], linear [10], and arthropathy [11]. Heterogeneous are also the neoplastic pathologies that can be associated with psoriasis induced by pembrolizumab. Indeed, there have been reported cases associated with lung [3], bladder [8], gastric [11], and cutaneous (melanoma [12,13]) cancers.

The risk of developing psoriasis with ICIs is known. Most cases show a psoriasis eruption in patients with pre-existing disease, but a new disease outbreak has been reported. The pathogenesis has not been clarified, but psoriasis is a chronic, immune-mediated disorder facilitated by cytotoxic T cells and, more specifically, by T helper (Th1) and Th17-cells. Interleukin (IL)-17 and IL-22 produced by Th1, 17 and 22 cells have an important role. T-cell activation induced by ICI facilitates the psoriasis onset or worsening in patients with personal or family history. It has been observed that the PD-1 axis downregulates the Th1/Th17 signaling pathway [14]. Moreover inhibiting this pathway, an upregulation of Th17 lymphocytes with secondary overexpression of proinflammatory cytokines is detected [15]. Therefore, Th17 upregulation could be the cause of this patient's psoriasis exacerbation during the antiPD-1 administration. The temporal connection between taking pembrolizumab and the onset of psoriasis in the patient's advanced age seems to us to be a good demonstration of the iatrogenic origin of the disease. Furthermore, the recurrence of psoriasis during treatment with pembrolizumab after discontinuation of apremilast demonstrates the close correlation between the events. Taking into account the probability scale of adverse drug reactions (Naranjo Scale), the value is 10. The reaction followed a reasonable temporal sequence after pembrolizumab, followed a recognized response to the suspected drug, and was confirmed by improvement on withdrawing the drug and reappeared on reexposure without apremilast. Treatments of pembrolizumab-induced psoriasis have included systemic corticosteroids, acitretin [3,12], and apremilast [7,13].

Our report is the second case described in the literature regarding pembrolizumab-induced rupioid psoriasis for neoplastic lung disease [3]. It could be that pembrolizumab induces the presence of a more intense psoriasiform reaction, such as the rupoid type, especially in those who have a personal or family history of psoriasis. Even the patient of the first case described in the literature had a positive personal history of psoriasis [3]. The rupioid reaction should be a pronounced inflammatory response, leading to abnormal keratinized scale mixing with copious sero-exudate [7].

However, while the previous case was undergoing therapy with acitretin and methylprednisone [3], our patient is the first case treated with apremilast with excellent and rapid remission even after discontinuation and re-administration of pembrolizumab without exacerbation of dermatitis.

## Conclusions

As far as we know, this is the second case of rupioid psoriasis induced by immunotherapy with pembrolizumab, and our experience demonstrates the good efficacy and safety of apremilast in such a clinical picture. In addition, the appearance of psoriasis during immunotherapy can be properly treated, and this does not contraindicate the continuation of the antineoplastic treatment. The reason for inducing inflammatory skin reactions by this antineoplastic drug is not yet known.
